# Nucleolin directly mediates Epstein-Barr virus immune evasion through binding to G-quadruplexes of EBNA1 mRNA

**DOI:** 10.1038/ncomms16043

**Published:** 2017-07-07

**Authors:** María José Lista, Rodrigo Prado Martins, Olivier Billant, Marie-Astrid Contesse, Sarah Findakly, Pierre Pochard, Chrysoula Daskalogianni, Claire Beauvineau, Corinne Guetta, Christophe Jamin, Marie-Paule Teulade-Fichou, Robin Fåhraeus, Cécile Voisset, Marc Blondel

**Affiliations:** 1 Institut National de la Santé et de la Recherche Médicale UMR1078; Université de Bretagne Occidentale, Faculté de Médecine et des Sciences de la Santé; Etablissement Français du Sang (EFS) Bretagne; CHRU Brest, Hôpital Morvan, Laboratoire de Génétique Moléculaire, 22 avenue Camille Desmoulins, Brest F-29200, France; 2 Cibles Thérapeutiques, Institut National de la Santé et de la Recherche Médicale UMR1162, Institut de Génétique Moléculaire, Université Paris 7, Hôpital St Louis, 27 rue Juliette Dodu, Paris F-75010, France; 3 Inserm UMR 1227, Lymphocytes B et Autoimmunité; Université de Bretagne Occidentale; CHRU Brest, Hôpital Morvan, Laboratoire d’Immunologie, Brest F-29200, France; 4 Chemistry, Modelling and Imaging for Biology, CNRS UMR9187 - Inserm U1196, Institut Curie, Université Paris-Sud, Campus universitaire, Bat. 110, Orsay F-91405, France

## Abstract

The oncogenic Epstein-Barr virus (EBV) evades the immune system but has an Achilles heel: its genome maintenance protein EBNA1, which is essential for viral genome maintenance but highly antigenic. EBV has seemingly evolved a system in which the mRNA sequence encoding the glycine-alanine repeats (GAr) of the EBNA1 protein limits its expression to the minimal level necessary for function while minimizing immune recognition. Here, we identify nucleolin (NCL) as a host factor required for this process via a direct interaction with G-quadruplexes formed in GAr-encoding mRNA sequence. Overexpression of NCL enhances GAr-based inhibition of EBNA1 protein expression, whereas its downregulation relieves the suppression of both expression and antigen presentation. Moreover, the G-quadruplex ligand PhenDC3 prevents NCL binding to EBNA1 mRNA and reverses GAr-mediated repression of EBNA1 expression and antigen presentation. Hence the NCL-EBNA1 mRNA interaction is a relevant therapeutic target to trigger an immune response against EBV-carrying cancers.

## Abstract (web summary)

Cells infected with Epstein-Barr virus (EBV) express the virus-encoded EBNA1, which is essential for viral genome maintenance but also highly antigenic. Here the authors implicate nucleolin as a host factor that mediates the repression of EBNA1-derived antigenic peptides through binding of the G4-quadruplex structure present within the EBNA1 mRNA.

The Epstein-Barr virus (EBV) was the first oncogenic virus discovered in human^1^,^2^,^3^ and has been linked to various cancers that include Burkitt and Hodgkin lymphomas and 10% of gastric cancers. Another example is the nasopharyngeal carcinoma, which is particularly frequent among men in China and Tunisia. Like all the gammaherpesviruses, EBV evades the host immune system but has an Achilles heel: its genome maintenance protein (GMP) EBNA1 (refs 4, 5). Indeed, EBNA1 is essential for EBV genome replication and maintenance and as such expressed in all dividing EBV-infected cells. EBNA1 is also highly antigenic and CD8^+^ T cells directed towards EBNA1 epitopes exist in all infected individuals. Hence, EBV has evolved a mechanism to limit EBNA1 production to the minimal level required for viral genome replication while keeping to a minimum the production of EBNA1-derived antigenic peptides presented to the cytotoxic T cells through the MHC class I pathway^4^,^6^. The central glycine-alanine repeat (GAr) of EBNA1 plays a critical role in this mechanism of immune evasion as it suppresses the translation of its own mRNA *in cis*^7^,^8^. The high level of EBNA1 protein and the efficient T cell response following the infection by an EBV strain encoding a truncated version of EBNA1 in which GAr has been deleted (EBNA1ΔGAr) demonstrates the critical role of GAr in EBNA1 immune evasion^7^,^8^,^9^,^10^. In line, a polymorphism in the length of GAr exists and, importantly, the effect of GAr is length-dependent as a longer domain displays a stronger inhibitory effect on both mRNA translation and antigen presentation^11^.

The GAr-encoding mRNA sequence is GC rich and forms predicted G-quadruplex (G4) structures that have been implicated in the regulation of EBNA1 synthesis *in vitro*^12^. G4 are particular secondary structures of nucleic acid formed by the stacking of G-quartets which correspond to a planar arrangement of four guanines connected by Hoogsteen hydrogen bonds. G4 structures within G-rich DNA or RNA sequences have been implicated in gene regulation where they can affect transcription, alternative splicing and translation^13^,^14^,^15^,^16^,^17^. G4 modes of action are not completely understood, but cellular factors that can interact with these structures are emerging^18^,^19^,^20^.

Nucleolin (NCL) is a multifunctional DNA/RNA-binding protein widely conserved among eukaryotes. It is involved in RNA metabolism, in particular in rRNA maturation^21^. NCL binds to G-rich sequences in coding and non-coding regions of various mRNA, many of which encode cancer-related proteins, and enhance their translation^22^. In addition, NCL binds to G4 structures within DNAs and RNAs. For example, NCL binds to and stabilizes G4 structures formed within the LTR promoter of HIV, thereby silencing the provirus transcription^23^. NCL also affects the transcription of c-MYC by binding to and stabilizing a G4 structure present in the promoter of this oncogene^17^,^24^.

GAr-based EBNA1 immune evasion has been considered a relevant therapeutic target against EBV-related cancers as most tumour cells from EBV-related cancers are infected by EBV and thus express EBNA1 at a minimal level. Importantly, the latent infection by EBV is primarily restricted to a specific small pool of memory B cells. Hence, overcoming GAr-based self-inhibition of EBNA1 translation should unveil EBV-carrying tumour cells to cytotoxic T cells without having a significant effect on the vast majority of non-tumour host cells. As neither the mechanisms of GAr-mediated mRNA translation suppression in *cis* nor the cellular factors involved are known, we developed a yeast-based (*Saccharomyces cerevisiae*) assay that recapitulates the key aspects of the GAr-based inhibition of translation, including length dependency^25^,^26^. This assay was previously used to identify small molecular weight compounds that can stimulate EBNA1 expression both in yeast and mammalian cells and that relieve GAr-based limitation of antigen presentation^26^.

Here, using this assay, we have isolated the yeast *NSR1* gene, which encodes the orthologue of human NCL, and show that NCL is a host factor required for GAr-based suppression of translation and to minimize antigen presentation. We also show that NCL directly interacts with G4 formed in the GAr-encoding sequence of EBNA1 mRNA. Finally, we show that this interaction is druggable, as the G4 ligand PhenDC3 prevents NCL from binding to G4 formed in the GAr mRNA sequence and stimulates EBNA1 expression and antigen presentation.

Hence, NCL is a host cell factor critically involved in EBNA1 immune evasion and the NCL-EBNA1 mRNA interaction appears to be a relevant therapeutic target to treat EBV-related cancers.

## Results

### *NSR1* mediates GAr effect on protein expression in yeast

**Figure 1:**
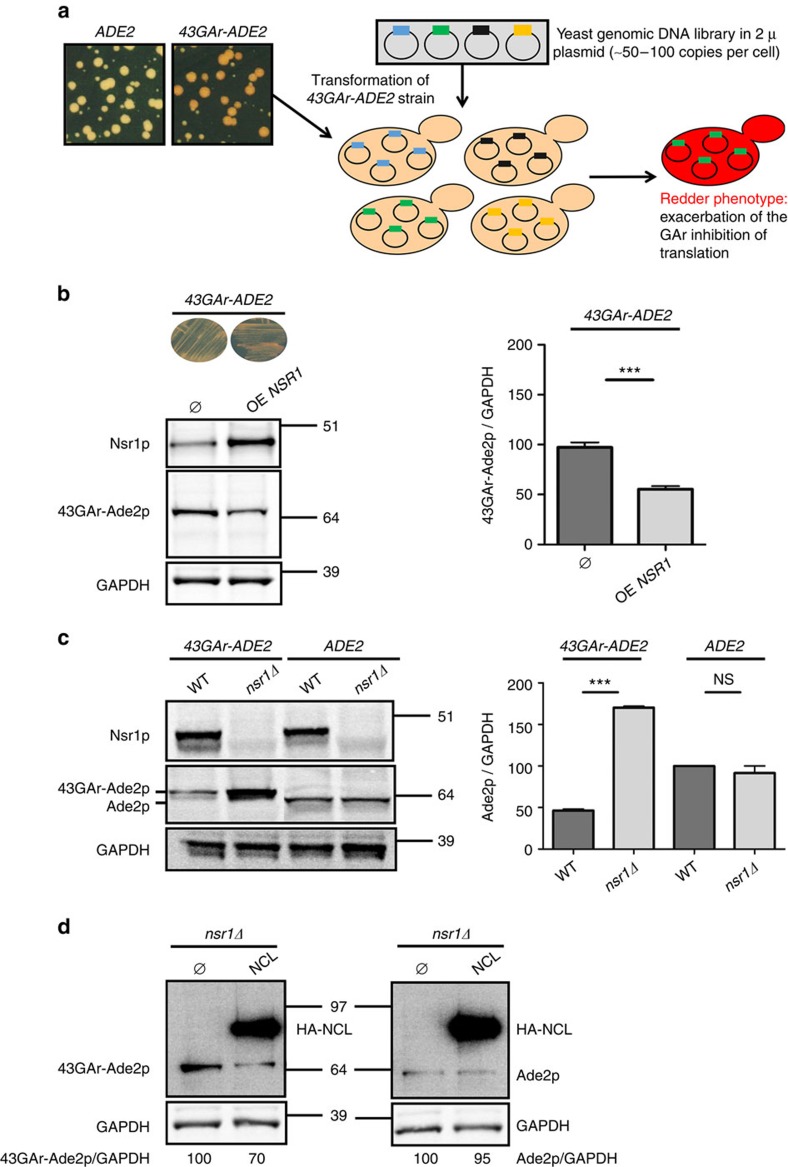
Identification and confirmation of the critical role of nucleolin in GAr-based translation inhibition in yeast. (**a**) Rationale of the yeast-based genetic screen. Contrary to yeast cells expressing *ADE2* gene (left panel) that form white colonies on rich medium (YPD), those expressing the *43GAr-ADE2* fusion (right panel) form pink colonies due to the ability of GAr to self-limit the translation of its own mRNA in yeast as in human cells. On the basis of this model, we looked for yeast genes whose overexpression from high copy number plasmids leads to a redder phenotype, suggesting an exacerbation of the GAr-based translation inhibition. (**b**) Effect of *NSR1* overexpression on 43GAr-Ade2p protein level. The overexpression of *NSR1* gene, which encodes the yeast nucleolin, led to a redder phenotype associated to a decrease in 43GAr-Ade2p level as evidenced by SDS-PAGE and western blot analysis (left panel). GAPDH was used as a loading control. The mean 43GAr-Ade2p/GAPDH ratio from three independent experiments is shown in the right panel and the results compared using the Student’s *t*-test. ***P<0.001. (**c**) Effect of *NSR1* gene deletion on 43GAr-Ade2p and Ade2p protein level. SDS-PAGE and western blot analysis showing that deletion of *NSR1* (*nsr1Δ*) had no effect on Ade2p level, whereas it strongly increased the level of 43GAr-Ade2p. GAPDH was used as a loading control. The mean 43GAr-Ade2p/GAPDH or Ade2p/GAPDH ratios from three independent experiments are shown in the right panel and the results compared using the Student’s *t*-test (***P<0.001; NS: not significant). (**d**) Human nucleolin (NCL) is able to complement *NSR1* deletion. SDS-PAGE and western blot analysis of the yeast *nsr1Δ* strain expressing 43GAr-Ade2p (left) or Ade2p (right) and overexpressing (right lanes), or not (left lanes), HA-tagged human nucleolin (HA-NCL). GAPDH was used as a loading control. The 43GAr-Ade2p/GAPDH or Ade2p/GAPDH ratio is indicated below the gels. Blots represent *n*≥3.

The yeast assay used in this genetic screen^26^ is based on a fusion between the yeast Ade2p reporter protein and a GAr domain of 43 amino acid (43GAr). Because GAr is able to self-inhibit the translation of its own mRNA in yeast, this leads to a reduction in Ade2p level. This can easily be monitored in yeast as cells which express Ade2p at a functional level form white colonies, whereas cells that do not express Ade2p readily form red colonies and any intermediate level of Ade2p leads to the formation of pink colonies whose intensity of coloration is inversely proportional to the level of Ade2p expressed. Hence, a yeast strain expressing the *43GAr-ADE2* construct from the constitutive *ADH* promoter forms pink colonies, whereas a control strain expressing *ADE2* from the same promoter forms white colonies (Fig. 1a). We used the *43GAr-ADE2* strain to identify yeast genes whose overexpression leads to a redder phenotype meaning that they potentially exacerbate GAr-based inhibition of translation. For this purpose we transformed the *43GAr-ADE2* yeast strain by a yeast genomic DNA library consisting of small genomic fragments (∼4 kb) cloned into a yeast 2 μ multicopy plasmid, which is present at ∼50–100 copies per yeast cell, hence potentially allowing to assess the effect of overexpressing the vast majority of yeast gene on GAr-based inhibition of translation (indeed, the few yeast genes larger than 4 kb may not be fully overexpressed using this library). This way, we isolated two independent clones bearing overlapping genomic fragments that, among a few other genes, contained the yeast *NSR1* gene. We then subcloned *NSR1* gene alone under the control of the strong constitutive *GPD* promoter in a low copy number vector (CEN) and confirmed its ability, when overexpressed, to both confer a redder phenotype and exacerbate the ability of GAr to decrease 43GAr-Ade2p protein expression (Fig. 1b), whereas having no significant effect on Ade2p protein in the control strain (Supplementary Fig. 1a) or on *43GAr-ADE2* or *ADE2* mRNA levels (Supplementary Fig. 1b).

We then determined the effect of *NSR1* downregulation on GAr-based inhibition of protein expression. As *NSR1* is not essential in yeast, we deleted this gene in both *43GAr-ADE2* and *ADE2* strains and observed that its absence abolished the GAr inhibitory effect on 43GAr-Ade2p expression (first two lanes of the western blot in Fig. 1c). This effect was GAr-dependent as *NSR1* deletion had no effect on Ade2p protein level in the control strain (last two lanes of the western blot in Fig. 1c). As controls, we checked that *NSR1* deletion had no significant effect on *43GAr-ADE2* and *ADE2* mRNA levels (Supplementary Fig. 1c).

### Human NCL can functionally replace Nsr1p in yeast

Next, we assessed the potential ability of NCL, the gene encoding the human NCL, to functionally complement the deletion of *NSR1* in yeast and found that the expression of NCL led to a decrease in 43GAr-Ade2p level, while having no effect on Ade2p level (Fig. 1d). Of note, the human NCL was as efficient as the yeast Nsr1p (Supplementary Fig. 1d).

Taken together, these results show that Nsr1p, the yeast orthologue of NCL, is required for the GAr-based inhibition of protein expression in yeast. As NCL, the human nucleolin, is able to fully complement the deletion of the yeast *NSR1* gene, this suggests that NCL represents a host cell factor important for the GAr-mediated self-limitation of EBNA1 expression in EBV-infected human cells.

### NCL also controls EBNA1 expression in EBV-infected cells

**Figure 2:**
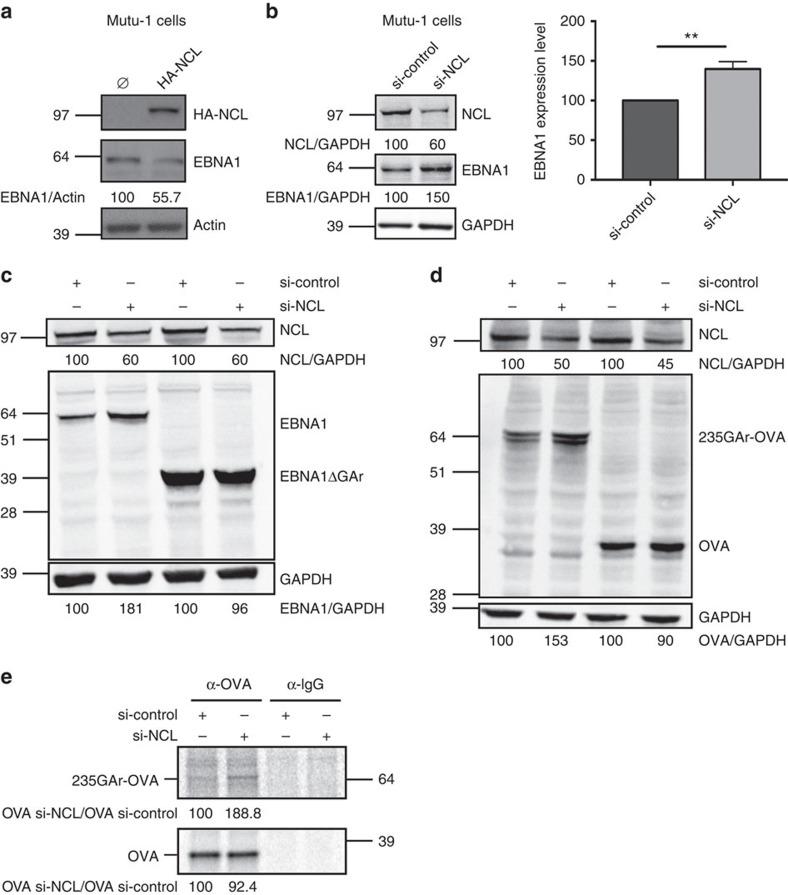
NCL also mediates GAr-based translation inhibition in human cells. (**a**) SDS-PAGE and western blot analysis of the level of endogenous EBNA1 in the EBV-infected B cell line Mutu-1 overexpressing (right lane), or not (left lane), HA-tagged nucleolin (HA-NCL). Actin was used as a loading control. EBNA1/actin ratios are indicated below the gels. Blot represents *n*≥3. (**b**) SDS-PAGE and western blot analysis of the level of endogenous EBNA1 in the Mutu-1 cells in response to NCL knockdown. Mutu-1 cells were transfected with control siRNA or siRNA targeting NCL, as indicated. GAPDH was used as a loading control. EBNA1/GAPDH ratios are indicated below the gels. The efficiency of NCL downregulation was estimated by determining the NCL/GAPDH ratio in cells treated by siRNA targeting NCL versus cells treated by control siRNA. The mean EBNA1/GAPDH ratio from three independent experiments is shown in the right panel and the results obtained with siRNA targeting NCL compared with control siRNA using the Student’s *t*-test (**P<0.01). (**c**) SDS-PAGE and western blot analysis of the level of EBNA1 or EBNA1ΔGAr in response to NCL knockdown. H1299 cells were transfected with EBNA1 or EBNA1ΔGAr expressing vectors and with control siRNA or siRNA targeting NCL, as indicated. GAPDH was used as a loading control. EBNA1/GAPDH or EBNA1ΔGAr/GAPDH ratios are indicated below the gels. The efficiency of NCL downregulation was estimated by determining the level of remaining NCL in cells treated by siRNA targeting NCL versus cells treated by control siRNA. Blot represents *n*≥3 and a representative result is shown. (**d**) Same experiment as in **c** except that H1299 cells were transfected with chicken ovalbumin (OVA) or 235GAr-OVA. Blot represents *n*≥3 and a representative result is shown. (**e**) Autoradiographs showing relative mRNA translation efficiencies of 235GAr-OVA versus OVA in response to NCL knockdown. H1299 cells transfected with 235GAr-OVA (upper panel) or OVA (lower panel) constructs and with control siRNA or siRNA targeting NCL, as indicated, were pulse-labelled with [^35^S] methionine, and lysates were subjected to immunoprecipitation with antibodies raised against OVA or IgG as a control, as indicated, and subjected to SDS-PAGE and autoradiography. Quantification of 235GAr-OVA or OVA signals are indicated.

We next assessed the role of human NCL on GAr-based self-inhibition of translation in human cells. For this purpose, we first overexpressed HA-tagged NCL (HA-NCL) in three EBV-infected B cell lines: Mutu-1, B95.8 and Raji. As shown in Fig. 2a for Mutu-1 and in Supplementary Fig. 2a for B95.8 and Raji, overexpression of HA-NCL led to a significant decrease in EBNA1 endogenous level in these three cell lines, as compared to actin. Similar to yeast, this effect is GAr-dependent as overexpression of HA-NCL also decreased the level of transfected 235GAr-OVA (ovalbumin), the fusion protein that is used to assess the effect of GAr on MHC class I-restricted antigen presentation (see below), whereas having no significant effect on OVA alone, the control protein (Supplementary Fig. 2b). The relative levels of endogenous EBNA1 and NCL were also determined in B95.8, Mutu-1 and Raji cells (Supplementary Fig. 2c).

We then determined the effect of downregulating endogenous NCL on endogenous EBNA1 level in EBV-infected Burkitt lymphoma Mutu-1 cells using siRNA. As observed by others^27^, we were not able to knockdown more than ∼40–50% the expression of NCL, probably because it is an essential gene in mammalian cells. However, this partial downregulation led to a significant increase (∼50%) in EBNA1 level (Fig. 2b) demonstrating that endogenous NCL expression restricts endogenous EBNA1 expression in EBV-infected cells.

To determine whether the impact of NCL level on EBNA1 expression is GAr-dependent, we then determined the effect of downregulating endogenous NCL on GAr-dependent suppression of protein expression in H1299 cells. Again, we were not able to knockdown more than ∼40–50% the expression of NCL. However, this partial downregulation led to a significant increase in both EBNA1 (Fig. 2c) and 235GAr-OVA (Fig. 2d) protein levels to about the same extent (∼80% and ∼50%, respectively) than the increase of endogenous EBNA1 in Mutu-1 cells, whereas having no effect on EBNA1ΔGAr or on OVA alone. Quantifications of three independent experiments are shown in Supplementary Fig. 2d,e.

Next, we performed a metabolic ^35^S methionine pulse-labelling experiment and we observed an increase in newly synthesized 235GAr-OVA following NCL downregulation (Fig. 2e, upper panel). In contrast, no increase in newly synthesized OVA was observed (Fig. 2e, lower panel). We did not observe any significant effect on the levels of GAr-carrying mRNAs (Supplementary Fig. 2f,g). Altogether, these results demonstrate that NCL downregulation does interfere with GAr-based suppression of translation.

These findings confirmed that, as in yeast, NCL represents a host cell factor critically involved in the GAr-dependent suppression of EBNA1 synthesis, a mechanism at the basis of EBV immune evasion in latently infected cells.

### NCL knockdown overrides GAr-restricted antigen presentation

**Figure 3:**
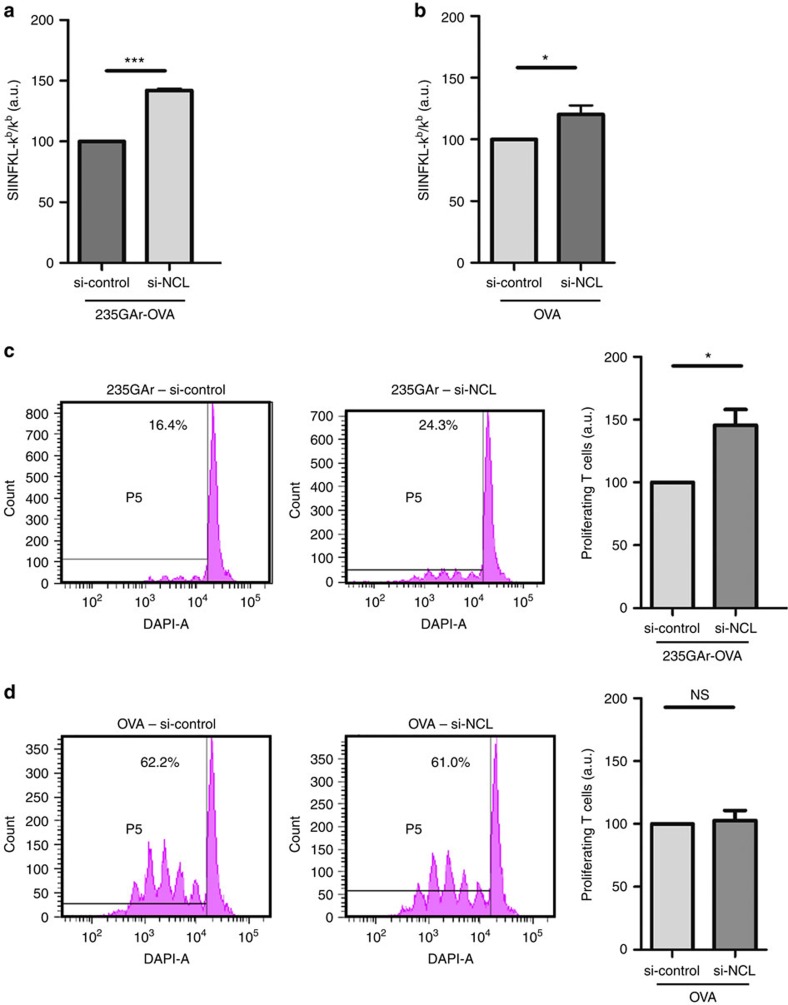
NCL downregulation activates GAr-limited antigen presentation and recognition by specific T lymphocytes. (**a**) Effect of NCL knockdown on antigen presentation. H1299 were transfected with 235GAr-OVA and murine MHC class I K^b^ plasmids and with control siRNA or siRNA targeting NCL, as indicated, and the levels of Kb/OVA-derived antigenic peptide complexes determined using FACS analysis (a.u.: arbitrary units). These experiments were performed three times and the mean quantification of the complex level obtained in cells treated by si-NCL or by si-control are shown and compared using the Student’s *t*-test (***P<0.001). (**b**) Same experiment as in **a** except that H1299 cells were transfected with OVA plasmid (*P<0.05). (**c**) Effect of NCL knockdown on T-cell proliferation. H1299 cells were transfected with mouse K^b^ and 235GAr-OVA plasmids and control siRNA (left) or siRNA targeting NCL (right). Afterwards cells were mixed with mouse naive OVA_257–264_ (SIINFEKL) specific CD8^+^ T-cells isolated from peripheral and mesenteric lymph-nodes and stained with CellTrace Violet. The proliferation of these SIINFEKL-specific T cells was determined by FACS analysis. Quantification of proliferating T lymphocytes when incubated in presence of cells treated by siRNA targeting NCL (si-NCL) or with control siRNA-treated cells (si-control) are shown on the right. The results were compared using the Student’s *t*-test (*P<0.05). (**d**) Same experiment as in **c** except that H1299 were transfected with OVA plasmid (NS: not significant).

We next determined if downregulating NCL has an effect on GAr-restricted MHC class I antigen presentation. Indeed, as NCL downregulation led to a GAr-dependent increase in protein expression, it was also expected to stimulate antigen presentation. For this purpose, we determined the effect of siRNA-mediated NCL knockdown on the GAr-restricted presentation of the ovalbumin-derived SIINFEKL antigenic peptide (OVA_257–264_) complexed with the murine k^b^ MHC class I receptor using a specific monoclonal antibody. FACS analysis revealed that NCL knockdown significantly increased (+42.1%±1.37, *P*=0.0005) the formation of this complex in 235GAr-OVA-expressing cells (Fig. 3a). In contrast, NCL knockdown only had a modest effect on OVA-expressing cells (+19.7%±6.11, *P*=0.043; Fig. 3b). In both cases, the efficiency of siRNA-mediated NCL downregulation and its effect on 235GAr-OVA or OVA expression were determined (Supplementary Fig. 3a).

We then tested whether the observed increase in antigen presentation following NCL downregulation does have an effect on T-cell activation. For this purpose, we determined the proliferation of naive CD8^+^ T cells (OT1 cells) recognizing specifically the OVA_257–264_ SIINFEKL epitope on the murine k^b^ MHC class I molecule. The OT1 cells were isolated from peripheral and mesenteric lymph-nodes of 12-week-old mice and stained with the CellTrace Violet fluorescent dye. Then, OT1 cells were mixed with H1299 cells expressing 235GAr-OVA and the k^b^ molecule. As a control, H1299 cells expressing OVA and the k^b^ molecule were used. As expected and due to the GAr inhibitory effect on both expression and antigen presentation, 235GAr-OVA-expressing H1299 cells led to a much weaker activation of OT1 cells (Fig. 3c, left panel) as compared to OVA-expressing H1299 cells (Fig. 3d, left panel), as determined by evaluating the number of dividing OT1 cells by FACS analysis. However, siRNA-mediated NCL knockdown in 235GAr-OVA-expressing H1299 cells significantly increased proliferation of OT1 cells (Fig. 3c, right panels), whereas it had no effect in OVA-expressing H1299 cells (Fig. 3d, right panels). The efficiency of siRNA-mediated NCL downregulation and its effect on 235GAr-OVA or OVA expression are shown in Supplementary Fig. 3b.

### NCL directly interacts with G4 of GAr-encoding mRNA

**Figure 4:**
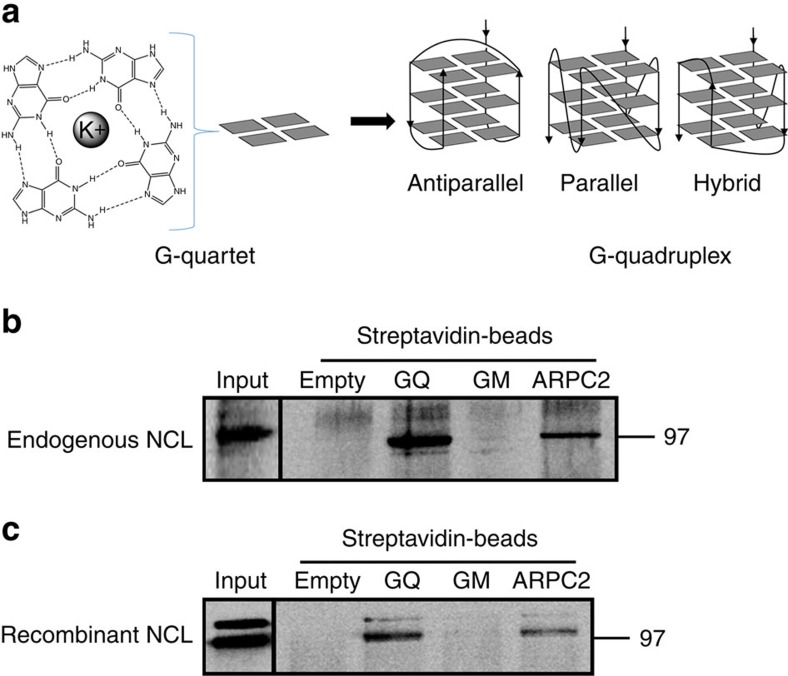
NCL directly interacts with G4 formed in the GAr-encoding mRNA. (**a**) Schematic representation of a G-quadruplexe (G4) structure. (Left) self-assembly of four guanines held together by Hoogsteen hydrogen bonds (dashed lines) giving a G-quartet in presence of K^+^ and schematic representation depicted by grey rectangles. Several G-quartets stack to form G4. (Right) the three main topologies adopted by G4 classified as function of strand orientation (indicated by arrows) and differing by loop arrangements. G4 RNA mostly adopt the parallel topology. (**b**) RNA pulldown using G4 forming RNA oligonucleotides covalently linked to biotin and streptavidin-coupled sepharose beads. Lysate from H1299 cells was applied to the following matrices: streptavidin-coupled beads either alone (Empty), or together with GQ (containing the most probable G4 of GAr mRNA), GM (same sequence except that G critical for G4 formation were replaced by adenines or uridines) or ARPC2 (containing a G4 present in *ARPC2* mRNA and that has been shown to bind NCL) RNA oligonucleotides. The sequence of these oligonucleotides is given in the Methods section. The proteins still bound to the beads after an 800 mM KCl wash were eluted and analysed by SDS-PAGE and western blot. (**c**) Same experiment as in **b** except that recombinant NCL was used instead of H1299 cell lysate.

NCL has been reported to bind to some G4 formed in both DNA^24^ and RNA sequences^28^. G4 are composed and stabilized by the stacking of guanine tetrads which are assembled in a planar arrangement by Hoogsteen hydrogen bonding (Fig. 4a) and have been involved in the regulation of gene expression, DNA replication and telomere maintenance 20. The G-rich sequence of GAr-encoding mRNA contains a cluster of 13 predicted G4 (ref. 29). Hence, we determined the ability of NCL to bind to these structures. For this purpose, we adapted a pulldown assay recently developed to identify RNA G4 binding proteins^28^ to an 18 nt-long oligonucleotide containing the most probable G4 that can form in the GAr-encoding mRNA sequence. Briefly, this oligonucleotide (GQ) was linked to biotin and pulldown experiments using streptavidin-conjugated sepharose beads were performed. As a negative control, we used an oligonucleotide (GM) with a similar sequence except that the four guanines forming the G4 were replaced by adenines or uridines to completely abolish the G4 structure, as predicted using the QGRS-H predictor software^30^. As a positive control, we used ARPC2 30 nt-long oligonucleotide which corresponds to a G4 found in the *ARPC2* mRNA and that has been shown to bind NCL^28^. As shown in Fig. 4b, NCL was precipitated from H1299 cell extracts when using GQ or ARPC2 oligonucleotides, but not when using GM or empty beads showing that NCL binds to G4 formed in the GAr mRNA sequence.

Next, we performed the same pulldown experiment using recombinant NCL instead of cell lysate. Similar results were obtained (Fig. 4c) showing that NCL directly binds GAr most probable G4.

**Figure 5:**
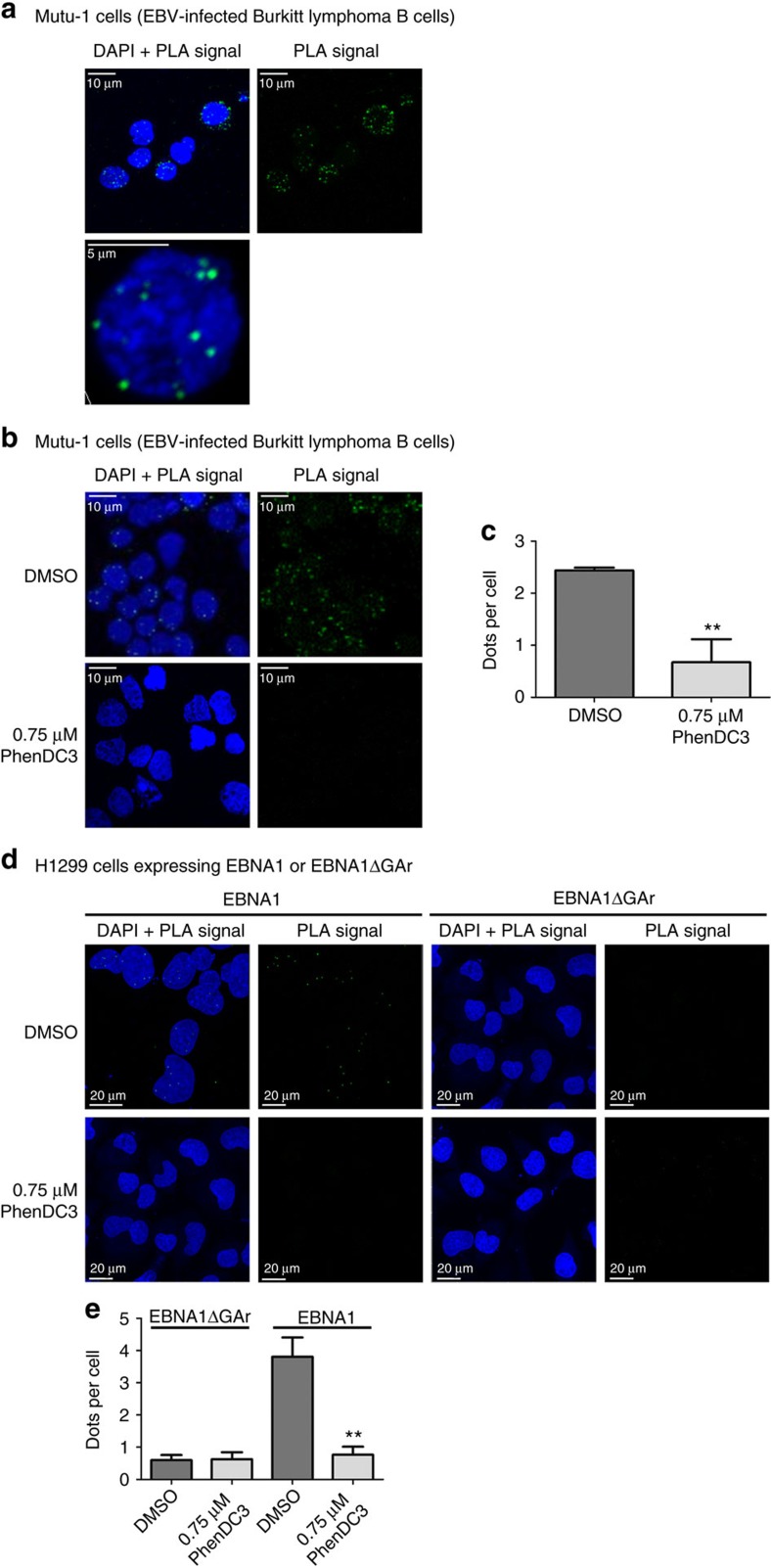
NCL-EBNA1 mRNA interaction takes place in the nucleus. (**a**) Endogenous NCL interacts *in cellulo* with endogenous EBNA1 mRNA in the nucleus. Proximity ligation assay (PLA) experiment to determine if NCL interacts with EBNA1 mRNA *in cellulo* was performed in the EBV-infected Burkitt lymphoma cells Mutu-1. The green dots (PLA signal) indicate an interaction. PLA signals were shown together with DAPI staining (top left) and alone (top right). A zoom on the nucleus of a Mutu-1 cell is shown. (**b**) *In cellulo* nuclear interaction between NCL and EBNA1 mRNA is reduced upon treatment by 0.75 μM PhenDC3. Same experiment than in **a** except that Mutu-1 cells were treated by DMSO (top) or PhenDC3 (bottom). (**c**) The mean number of PLA dots per cell in DMSO- (vehicle) or 0.75 μM PhenDC3-treated Mutu-1 treated cells shown in **b** are indicated and compared using the Student’s *t*-test (**P<0.01). (**d**) *In cellulo* nuclear interaction between NCL and EBNA1 mRNA is GAr-dependent. PLA experiments were performed in H1299 cells transfected with EBNA1 or EBNA1ΔGAr plasmids and treated or not with 0.75 μM PhenDC3. The green dots indicate an interaction. PLA signals were shown together with DAPI staining and alone. (**e**) The number of PLA dots per cells obtained in (**d**) treated by DMSO (vehicle) or 0.75 μM PhenDC3 are indicated and compared using the Student’s *t*-test (**P<0.01).

Finally, we wanted to verify if, and where, the NCL-GAr G4 interaction occurs *in cellulo*. Hence, we performed a proximity ligation assay (PLA) to assess if endogenous NCL associates with endogenous EBNA1 mRNA in EBV-infected Mutu-1 cells. We observed PLA dots mostly in the nucleus or close to it (Fig. 5a) indicating that endogenous NCL does interact with endogenous EBNA1 mRNA and that this interaction mostly takes place in the nucleus and in the cytoplasm close to the nucleus. In contrast, no PLA dots were observed in the various controls (without the probe specific for EBNA1 mRNA or without antibodies). To assess whether this interaction is GAr-dependent, we repeated this PLA in H1299 cells expressing EBNA1 or EBNA1ΔGAr. Again, we observed PLA dots in cells expressing the full-length EBNA1 mRNA (Fig. 5d,e). In contrast, almost no dots were detected in cells expressing EBNA1ΔGAr (Fig. 5d,e) indicating that the ability of NCL to interact with EBNA1 mRNA is GAr-dependent.

Taken together, these results indicate that NCL directly interacts with the GAr’s G4 of the EBNA1 mRNA and that this interaction mostly takes place in the nucleus or in the cytoplasm in the vicinity of the nucleus.

### PhenDC3 prevents GAr-based inhibition of protein expression

**Figure 6:**
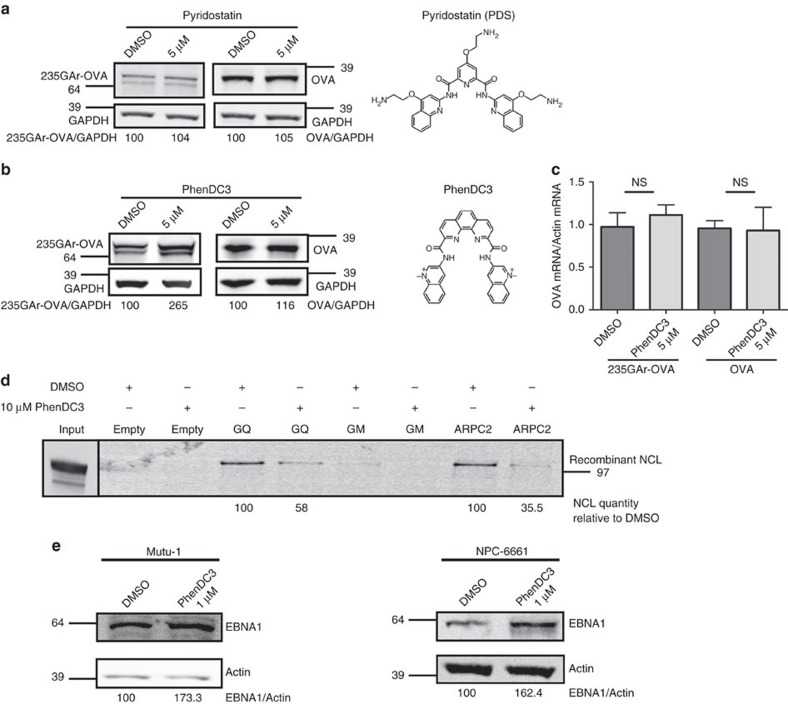
PhenDC3 prevents GAr inhibition of protein expression and NCL binding to GAr’s G4. (**a**) SDS-PAGE and western blot analysis of the level of 235GAr-OVA or OVA in response to pyridostatin (PDS) treatment. H1299 cells transfected with 235GAr-OVA (left) or OVA (right) plasmids were treated with 5 μM PDS (right lanes) or, as a control, with DMSO (left lanes). GAPDH was used as a loading control and 235GAr-OVA/GAPDH and OVA/GAPDH ratios are indicated below the gels. The chemical structure of PDS is depicted on the right. Blot represents *n*≥3. (**b**) SDS-PAGE and western blot analysis of the level of 235GAr-OVA or OVA in response to PhenDC3 treatment. Same experiments as in **a** except that cells were treated with 5 μM PhenDC3 which chemical structure is depicted on the right. Blot represents *n*≥3. (**c**) The effect of 5 μM PhenDC3 treatment on 235GAr-OVA or OVA mRNA level was determined by qRT-PCR. The results were compared using the Student’s *t*-test (NS: not significant). (**d**) PhenDC3 competes for the binding of NCL on GAr and *ARPC2* G4. Same experiment than in Fig. 4c in the presence of 10 μM PhenDC3 or DMSO (vehicle) as indicated. (**e**) PhenDC3 increases endogenous EBNA1 expression in EBV-infected cells. The level of endogenous EBNA1 in Mutu-1 (EBV-infected B cells, left panel) and NPC-6661 (EBV-infected cells from nasopharyngeal carcinoma, right panel) cells in response to 1 μM PhenDC3 was determined by SDS-PAGE followed by western blot. Actin was used as a loading control and EBNA1/Actin ratio are indicated below the gels. Blots represent *n*≥3.

Next, we tested the effect of various G4 ligands on GAr-based inhibition of protein expression. Among the reported G4 ligands, pyridostatin (PDS, molecular structure depicted in Fig. 6a) and PhenDC3 (molecular structure depicted in Fig. 6b) (refs 31, 32, 33) are the best benchmark probes compatible with cellular assays. Indeed, PDS and PhenDC3 at micromolar concentrations have been shown to efficiently target various G4 in cell-based experiments^34^. As, at the same range of concentrations (1–5 μM), PDS has been shown to exacerbate GAr-based inhibition of protein expression in an *in vitro* coupled transcription-translation assay^29^, we first tested the effect of this molecule on the level of 235GAr-OVA or OVA in H1299 cells. However, at the same concentration (5 μM), no clear effect on 235GAr-OVA or OVA expression was observed (Fig. 6a) suggesting that PDS may not be able to interfere with GAr-mediated inhibition of EBNA1 expression in a cellular context. We then tested PhenDC3 at the same concentration (5 μM) and found that it led to a significant increase in the steady-state level of 235GAr-OVA in H1299 cells (Fig. 6b, left panel). This effect is GAr-dependent since PhenDC3 had no significant effect on OVA expression (Fig. 6b, right panel) and is not due to an effect on the level of the corresponding RNA (Fig. 6c). Hence, one possibility is that PhenDC3 prevents the binding of NCL on EBNA1 mRNA G4.

### PhenDC3 prevents NCL-EBNA1 mRNA interaction

To test this hypothesis, we performed the same G4 oligonucleotide pulldown assay as in Fig. 4c in the presence or absence of 10 μM PhenDC3 and we observed that PhenDC3 does prevent the binding of NCL on GAr G4 (Fig. 6d), readily explaining its effect on 235GAr-OVA expression. Of note, PhenDC3 also prevents the binding of NCL on ARPC2 G4 (Fig. 6d). In contrast, PDS had no effect on the binding of NCL on both types of G4 (Supplementary Fig. 4a). We also checked that PDS and PhenDC3 are both able to bind to the most probable G4 that can form in the GAr-encoding mRNA sequence by determining the ability of these two compounds to displace the thiazole orange (TO) fluorescent probe from the GAr’s G4 oligonucleotide used in the pulldown experiments^35^. As shown in Supplementary Fig. 4b, both PhenDC3 and PDS bind the GAr’s G4 but the affinity of PhenDC3 (DC_50_ ∼0.26 μM) is higher than that of PDS (DC_50_ ∼0.47 μM). Taken together these results suggest that PhenDC3, but not PDS, is also able to prevent the binding of NCL on these G4 structures by a competitive mechanism. This difference, which may be due, at least in part, to the lower affinity of PDS for GAr’s G4, is consistent with the fact that PhenDC3 does interfere with the GAr self-inhibitory effect on protein expression whereas PDS is inactive.

To confirm that PhenDC3 prevents the binding of NCL on EBNA1 mRNA in more physiological settings, we tested the effect of PhenDC3 in the PLA experiment on EBV-infected Mutu-1 cells. As shown in Fig. 5b,c, the number of nuclear PLA dots per cell was significantly reduced (∼threefold) when Mutu-1 cells were treated with 0.75 μM PhenDC3 confirming the ability of PhenDC3 to interfere with this interaction in a cellular context. The same result was obtained when using H1299 cells expressing transfected EBNA1 (Fig. 5d,e).

### PhenDC3 increases EBNA1 expression in EBV-infected cells

Next, we tested PhenDC3 effect on endogenous EBNA1 expression in EBV-infected cells and found that it also increased the endogenous EBNA1 level in Mutu-1 (EBV-infected B-cells; Fig. 6e left panel) and NPC-6661 (EBV-infected cells derived from a nasopharyngeal carcinoma^36^; Fig. 6e right panel) cells. Importantly, PhenDC3 had no effect on EBNA1 mRNA level in Mutu-1 cells (Supplementary Fig. 4c). We also checked that PhenDC3 is not significantly toxic on Mutu-1 cells when used at a concentration range (0.5–1 μM) in which it increases the expression of EBNA1 (Supplementary Fig. 4d). Finally, we found that PDS had no effect on endogenous EBNA1 level in Mutu-1 cells (Supplementary Fig. 4e) confirming that, contrary to PhenDC3, PDS is not able to interfere with the GAr-based self-inhibition of protein expression.

To conclude, the PhenDC3 G4 ligand prevents the binding of NCL on GAr’s G4 and, at the same time, leads to an increase in EBNA1 and 235GAr-OVA expression, thereby supporting the crucial role of NCL in GAr-based self-inhibition of translation by binding to G4 formed in the EBNA1 mRNA. Importantly, these results also indicate that NCL-EBNA1 mRNA interaction is druggable.

### PhenDC3 activates GAr-limited antigen presentation

**Figure 7:**
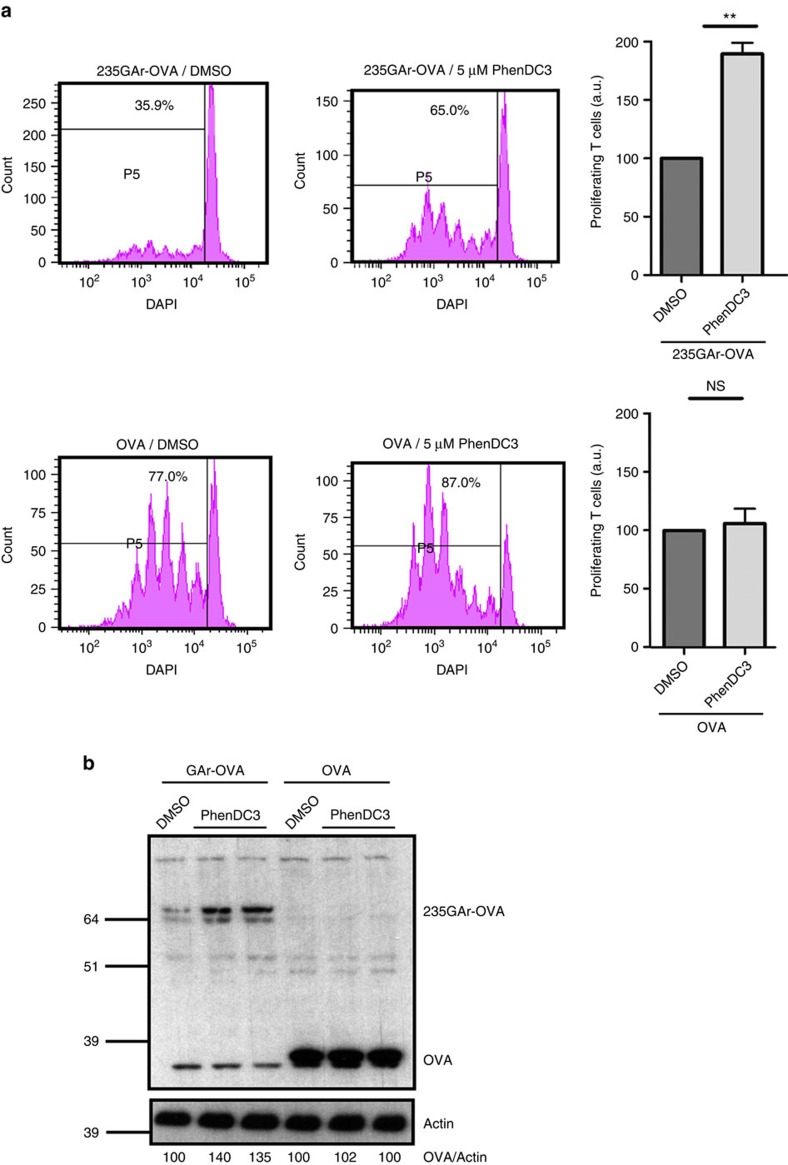
PhenDC3 activates GAr-limited antigen presentation. (**a**) PhenDC3 increases T-cell proliferation. Same experiment as in Fig. 3c,d except that 235GAr-OVA (upper panels) and OVA (lower panels) expressing H1299 cells were treated with 5 μM PhenDC3 or, as control, with DMSO as indicated. Quantifications of proliferating T lymphocytes following PhenDC3 treatment as compared to DMSO-treated cells are shown in the graphs on the right. The results were compared using the Student’s *t*-test (**P<0.01; NS: not significant). (**b**) SDS-PAGE and western blot analysis of cells used in **a**.

Finally, to assess the ability of PhenDC3 to interfere with GAr-based immune evasion, we performed the same OT1 T-cell proliferation assay as in Fig. 3c,d in the presence or absence of 5 μM PhenDC3. As shown in Fig. 7a, PhenDC3 significantly increased (twofold change) the proliferation of OT1 T cells added to 235GAr-OVA-expressing H1299 cells, whereas it had no effect on OT1 cells incubated with OVA-expressing cells. A western blot analysis confirmed that PhenDC3 increases the level of 235GAr-OVA, whereas it has no effect on OVA (Fig. 7b).

## Discussion

In this study, we have identified NCL as a host cell factor critically involved in GAr-based EBNA1 immune evasion by its ability to bind G4 formed in the GAr-encoding sequence of the EBNA1 mRNA. In line with this model, we showed that the G4 ligand PhenDC3 both prevents the binding of NCL on G4 of the EBNA1 mRNA, and increases EBNA1 expression and GAr-dependent antigen presentation. This indicates that the interaction between NCL and G4 of the EBNA1 mRNA is a relevant and druggable therapeutic target to treat EBV-related cancers. Interestingly, the G4 ligand PDS had no effect on EBNA1 expression *in cellulo* indicating that only some G4 ligands are able to interfere with NCL-EBNA1 mRNA interaction, which can be attributed to off-target binding and/or differences in pharmacological properties (cell penetration, intracellular distribution and so on). Of note, *in vitro*, PDS affinity for GAr’s G4 appears significantly weaker than that of PhenDC3 which may contribute, at least in part, to the differential activity of these two G4 ligands on GAr. This also points out two different possible mechanisms of action for G4 ligands whose binding on G4 may either stabilize them or prevent the binding of cellular partners by direct competition or by modifying G4 structure. In line with these hypotheses, we tested several PhenDC3 close chemical derivatives and observed that some, but not all, were active on EBNA1 expression (data not shown). Of note, PDS has been found to suppress EBNA1 expression in an *in vitro* coupled transcription-translation assay^29^ but it is not known, in this experiment, if this effect is related to a change in EBNA1 mRNA level or to G4 stabilization that may exacerbate the GAr-dependent translation inhibition.

This brings out an intriguing point regarding the role of NCL in GAr-based self-limitation of EBNA1 expression and antigenic presentation. Indeed, NCL has been also positively involved in EBV episome maintenance and transcription^27^. Hence, NCL appears to positively control both EBV episome maintenance and transcription on the one hand and the self-limitation of the EBV GMP expression on the other. As for EBV, one can consider it makes sense to have the same host cell protein regulating these two key aspects of EBV’s latency. Indeed, if NCL level is low, then the maintenance and transcription of EBV episome should be compromised but, as a result of NCL role in GAr-based limitation of EBNA1 expression, EBNA1 mRNA should be more efficiently translated, which may compensate for its reduced level and favour the maintenance of EBV genome. On the contrary, if NCL level is high, then EBV episome will be efficiently maintained and transcribed, hence leading to a high level of EBNA1 mRNA, but then the increased NCL could further downregulate its translation, thereby limiting the level of EBNA1 protein and therefore its detection by the immune system. Importantly, the role of NCL in EBNA1 immune evasion involves its ability to interact with G4 structures present in EBNA1 mRNA, whereas its role in episome maintenance and transcription involves its ability to interact with EBNA1’s N-terminal 100 amino acids (hence upstream of the GAr domain of EBNA1 protein). Therefore, targeting the NCL-EBNA1 mRNA interaction should specifically affect EBNA1 immune evasion.

What could be NCL mechanism of action in GAr-based self-inhibition of translation and antigen presentation? EBNA1 G4 may constitute a recognition signal for NCL that is, itself, directly or indirectly, responsible for translation inhibition by interfering with either translation initiation and/or elongation machinery. Alternatively, NCL could stabilize G4 that, themselves, may inhibit the ribosome progression. In either case, it is unlikely that the virus has developed a novel mechanism to exploit NCL for controlling gene expression. Rather, it is likely that this reflects a more general evolutionary conserved cellular pathway. The fact that NCL effect on GAr-based limitation of protein expression is also operant in yeast strengthens this hypothesis as yeast has no common evolutionary history with EBV. Hence, PhenDC3 and more generally G4 ligands represent chemical probes that will greatly help to characterize this pathway and to identify its physiological mRNA targets. To finish, as other oncogenic gamma herpesviruses like the Kaposi sarcoma-associated herpesvirus have evolved similar strategy of G4 clustering in the central coding regions of their GMP to evade the immune system, the results presented here may have applications for other gammaherpesviruses-related diseases^29^,^37^.

## Methods

### Yeast strains and culture media

All the yeast strains used in this study are derived from the W303 WT strain^38^: *MATa*, *leu2-3,112 trp1-1 can1-100 ura3-1 ade2-1 his3-11,15*. The *ade2Δ* strain genotype is: *MATa*, *leu2-3,112 trp1-1 can1-100 ura3-1 ade2-1:his5 S. pombe*. Yeast cells were grown and used as previously described^39^. The media used for yeast growth were: YPD (1% (w/v) yeast extract, 2% (w/v) peptone, 2% (w/v) glucose), 1/3 YPD (0.33% (w/v) yeast extract, 2% (w/v) peptone, 2% (w/v) glucose). Yeast minimal media w/o uracil (6.7% (w/v) yeast nitrogen base, 0.77% (w/v) amino acids without uracil, 2% (w/v) glucose). Yeast minimal media w/o uracil and tryptophan (6.7% (w/v) yeast nitrogen base, 0.72% (w/v) amino acids without uracil, 2% (w/v) glucose). Solid media contained 2% (w/v) agar.

### Yeast-based genetic screen that allowed isolation of *NSR1*

A yeast genomic DNA library (a kind gift by F. Lacroute) constructed by inserting ∼4 kb genomic DNA fragments (obtained by *Sau3A* partial digestion) at the unique BamHI site in the replicative 2 μ multicopy pFL44L vector containing *URA3*-marker, was transformed into 43GAr-Ade2p pink yeast strain using standard lithium acetate procedures^40^. This multicopy plasmid is present at ∼50–100 copies per yeast cell. Transformants were selected on uracil-free minimal solid medium and a positive selection was carried out based on the redder colour phenotype. Out of ∼20,000 transformants growing on uracil-free medium, 39 gave a redder phenotype. Plasmids originated from the pFL44-based library were extracted from these 39 redder transformants, purified and amplified in *Escherichia coli* and then retransformed into 43GAr-Ade2p yeast strain for confirmation of the redder phenotype. The extremities of the confirmed clones were sequenced using the following primers: F- 5′ GTGCTGCAAGGCGATTAAGT 3′ and R- 5′TGTGGAATTGTGAGCGGATA 3′. Two confirmed clones contained overlapping genomic fragments bearing the yeast *NSR1* gene.

### Plasmid constructions

All vectors were constructed using standard cloning procedures. T4 DNA ligase and restriction enzymes were purchased from New England Biolabs. Plasmid maintenance was carried out in TOP10 *E. coli* strain. The p416 (*GPD*) containing *NSR1* gene was constructed as follows: *NSR1* coding sequence was amplified from genomic DNA of the *S. cerevisiae* W303 WT strain using the following primers: NSR1-F 5′ CGCGGATCCATGGCTAAGACTACTAAAGTAAAAGGTAAC 3′ and NSR1-R 5′ CCGCTCGAGCGGTTAATCAAATGTTTTCTTTGAACCAG 3′. The corresponding PCR fragment was cloned into *BamHI* and *XhoI* cloning sites of p416 (*GPD*) centromeric vector. To introduce a HA tag in frame with human *NCL*, its coding sequence was PCR-amplified from cDNA extracted from HEK293T cells using HA-NCL F- 5′ CGCGGATCCATGTACCCATACGATGTTCCAGATTACGCTGTGAAGCTCGCGAAGGCAG 3′ and NCL R- 5′ CCGCTCGAGCGGCTATTCAAACTTCGTCTTCTTTCC 3′ primers and cloned into pCDNA3 vector (invitrogen) using *BamHI* and *XhoI* cloning sites. HA-NCL was then subcloned into the *S. cerevisiae* vector p414 (*GPD*). All generated constructs were amplified in the TOP10 *E. coli* strain, and sequenced by the Sanger method.

### Generation of *nsr1Δ* yeast strains

*NSR1* gene deletion was carried out by replacement with kanMX6 cassette amplified from PFA6a-kanMX6 vector^41^, using the following primers: F-5′ ACCAATTTCGGATCACTCAACCCAGGCAGGATAAAATAAGCGGATCCCCGGGTTAATTAA 3′ and R-5′ AAGAGAAAAAATTGAAATTGAAATTCATTTCATTTTCTCAGAATCCGAGCTCGTTTAAAC 3′. Then the PCR fragment was transformed into W303 *ade2*Δ, *43GAr-ADE2* and W303 *ade2*Δ, *ADE2* yeast strains using standard lithium acetate procedures^40^. The transformed cells were spread on YPD+100 μg ml^−1^ kanamycin plates which were then incubated 5 days at 29 °C, after which the plates were replicated on fresh YPD+100 μg ml^−1^ kanamycin plates, and the deletion of *NSR1* gene in kanamycin-resistant colonies was checked by PCR, using the following primers: *nsr1Δ* Fbis-5′ GTACTTAAGTGTAGCTGTTGC 3′ and *nsr1Δ* Rbis-5′ TAGAGATGGTGAATGAAAGG 3′.

### Yeast protein extracts

Five millilitres of 0.8–1.0 OD_600_ _nm_ exponentially growing cells was collected and cell pellets were resuspended into 300 μl of lysis buffer (25 mM Tris-HCl pH 6.8; 10% glycerol; 5% β-mercaptoethanol; 5% SDS; 8 M Urea; 0.02% bromophenol blue).

### Mammalian cells protein extracts

Whole cells were collected 48 h post transfection and lysed in 20 mM Tris-HCl, pH 7.5, 150 mM NaCl, 1% Igepal containing protease inhibitors (Roche, Germany). Samples were centrifuged at 16,000*g* for 20 min at 4 °C and protein concentrations were measured using a Bradford assay.

### Western blotting

Equal protein quantities and volumes of all samples were loaded onto 10% NuPAGE Bis-Tris gels (invitrogen), and transferred onto 0.45 μm nitrocellulose membranes (GE Healthcare). Membranes were blocked during 1 h at room temperature in PBS 1X containing 0.1% Igepal and 3% BSA.

Membranes were analysed using the following antibodies: anti-HA rat monoclonal antibody (Roche, 1:2,500); anti-Nsr1p mouse monoclonal antibody (Abcam, 1:5,000), anti-NCL rabbit polyclonal antibody (Abcam, 1:5,000), anti-GAPDH (Sigma, 1:5,000), anti-EBNA1 mouse monoclonal antibody (OT1X, 1:2,000), anti-OVA rabbit polyclonal antibody (Sigma, 1:2,500), anti-actin (Sigma, 1/5,000). The membranes were then washed with fresh PBS 1X+0.1% Igepal and incubated for 45 min with swine anti-rabbit or goat anti-mouse secondary antibodies (Dako) conjugated to horseradish peroxidase at a 1:3,000 dilution, and analysed by enhanced chemiluminescence (ECL, GE Healthcare) using a Vilbert-Lourmat Photodocumentation Chemistart 5000 imager. All the experiments were repeated at least three times. Relative protein levels for each sample were normalized to GAPDH or Actin protein levels as indicated, using Fusion-Capt Advance software.

### Cell culture and transfection

H1299 cells are derived from metastatic lymph node from lung carcinoma. Raji cells are type III latency Burkitt’s lymphoma. HCT116 cells are derived from colorectal carcinoma. B95.8 cells are derived from cotton-top Tamarin Monkey peripheral blood lymphocyte. Mutu-1 cells are derived from an EBV-positive Burkitt’s lymphoma biopsy specimen from a Kenyan patient. NPC-6661 cell line was established from a xenografted NPC in the early 1990s (ref. 36) and was kindly provided by Prof. Kwok-Wai Lo from the Chinese University of Hong Kong. H1299, Raji, B95.8 and Mutu-1 cells were cultured in RPMI-1640 media supplemented with 10% fetal bovine serum (FBS) and 2 mM L-glutamine. HCT116 cells were cultured in McCoy’s 5A Glutamax media supplemented with 10% FBS, and NPC-6661 cells in RPMI-1640 media supplemented with 25 mM HEPES (Gibco) and 2 mM L-glutamine and 10% FBS. All cells were cultured at 37 °C with 5% CO_2_. Transient transfections were performed using Genejuice reagent (Merck Bioscience) according to the manufacturer’s protocol or electroporation using Gene PulserXL system (Biorad).

### RNA extraction and quantitative real-time PCR

Total yeast, H1299 or Mutu-1 cellular RNA was extracted using RNAeasy and RNAase-free, DNase kits (Qiagen). cDNA synthesis was carried out using 1 μg of DNA-free RNA using M-MLV reverse transcriptase (invitrogen) and Oligo-dT primer. Triplicated cDNA samples were analysized by quantitative PCR using PERFECTA SYBR fastmix (Quanta Bioscience). The relative abundance of target mRNA was normalized using Actin as an endogenous control. Quantification of gene expression was determined using the –2^ΔΔCt^ method. The primers used for PCR were *ADE2*-forward: 5′-ATTGTGCAAATGCCTAGAGGTG-3′, *ADE2*-reverse: 5′-AATCATAA -GCGCCAAGCAGTC-3′; Actin-forward: 5′-ATGGTNGGNATGGGNCARAAR-3′, Actin-reverse: 5′-CTCCATRTCRTCCCAGTTGGT-3′; *EBNA1*-forward: 5′-GGCAGTGGACCTCAAAGAAGAG-3′; *EBNA1*-reverse: 5′-CAATGCAACTTGGACGTTTTT-3′; OVA-forward: 5′-GAGGAGGCTTGGAACCTAT-3′; OVA-reverse: 5′-CAGTTTGAGAATCCACGGAG-3'. All the experiments were performed in triplicates and were repeated at least three times.

### NCL siRNA downregulation

1.75 × 10^5^ H1299 cells were transfected with 0.75 μg of EBNA1, EBNA1ΔGAr, 235GAr-OVA or OVA vectors using standard procedures and incubated at 37 °C for 8 h. Cells were then transfected either with 40 nM of control siRNA or FlexiTube GeneSolution for NCL (Qiagen). siRNA transfection were performed using HiPerFect transfection reagent (Qiagen) following the manufacturer’s protocol. Forty hours after siRNA transfection, cells were collected for western blot or flow cytometry analyses.

Mutu-1 cells were electroporated using SG Cell Line 4D-Nucleofector X Kit from Lonza (V4XC-3012) following manufacturer’s instructions and 300 nM of control siRNA or FlexiTube GeneSolution for NCL (Qiagen). Forty hours after siRNA transfection, cells were collected for western blot analyses.

### Flow cytometry analysis

Forty-eight hours after the transfection, cells were collected using trypsin and washed twice with 1X PBS. Cells were suspended in 50 μl of 1X PBS and incubated with 0.4 μg of anti-mouse OVA257–264 (SIINFEKL) peptide antibody bound to H-2 Kb PE or anti-mouse MHC Class I (H-2 Kb) antibody bound to PE (Ebioscience) for 30 min at room temperature. Cells were then washed with 1X PBS and analysed by FACS on a CANTO II flow cytometer (BD Biosciences, USA).

### RNA pulldown experiments

For the preparation of whole cell extracts, confluent H1299 cells were collected after trypsin treatment and washed twice with 1X PBS (Gibco). Cells were suspended in 500 μl of lysis buffer (20 mM Tris-HCl pH 7.5; 200 mM NaCl and 0.1% Igepal) containing 1X protease inhibitor cocktail (Roche). Cell lysis was performed by five series of vortex followed by 10 min incubation on ice, and three series of 3 s sonication at 20% amplitude. After lysis cells were centrifuged at 4 °C for 15 min at 16,000*g*, and the supernatant was quantified by Bradford. The whole cell extracts or recombinant GST-NCL (Abnova) were used for pulldown assays with the following G-quadruplex forming oligonucleotides: GQ- 5′-GGGGCAGGAGCAGGAGGA-3′Biotin TEG, ARPC2- 5′ AGCCGGGGGCUGGGCGGGGACCGGGCUUGU-3′Biotin TEG. The negative control for EBNA1 G4 was the GM- 5′ GAGGCAGUAGCAGUAGAA-3′Biotin TEG oligonucleotide which, according to the GQRS-H predictor software^30^, is unable to form G4 structures. To avoid unspecific binding, high-affinity streptavidin sepharose beads (GE Healthcare) were incubated in 1 ml blocking buffer containing 10 mM Tris-HCl pH 7.5; 100 mM KCl; 0.1 mM EDTA; 1 mM DTT; 0.01% Triton X-100; 0.1% BSA; 0.02% *S. cerevisiae* tRNAs (Sigma), for 1 h at 4 °C on a rotating wheel. An amount of 10 pg of each folded biotinylated RNA oligos was incubated with 50 μl of solution containing the streptaviding sepharose beads for 90 min at 4 °C on a rotating wheel. Five hundred micrograms of cell extract or 200 ng of recombinant GST-NCL (Abnova) was incubated with the RNA oligonucleotides bound to the streptavidin beads during 90 min at room temperature. Beads were washed with increasing KCl concentration (200–800 mM). Protein still bound to beads after the washes were eluted using 2X SDS loading buffer and analysed by western blotting against NCL, as previously described. In the input lane of the western blots was loaded a quantity of extract which corresponds to half of the quantity that was incubated with the beads for each condition.

### Proximity ligation assay

Cells were fixed with 4% paraformaldehyde in PBS 1X for 20 min and permeabilized with 0.4% Triton X-100, 0.05% CHAPS for 5 min at room temperature. A quantity of 50 ng of EBNA1-digoxigenin mRNA probe (5′ CTTTCCAAACCACCCTCCTTTTTTGCGCCTGCCTCCATCAAAAA 3′) or control sense probe was denaturated 5 min at 80 °C and the hybridization reaction was carried out overnight at 37 °C in 40 μl hybridization buffer (10% formamide; 2X SSC, 0.2 mg ml^–1^ *E. coli* tRNAs, 0.2 mg ml^–1^ sheared salmon sperm DNA and 2 mg ml^–1^ BSA. A blocking solution of 3% BSA 0.1% saponine in 1X PBS was added for 30 min followed by 2 h incubation at room temperature with the primary antibodies (anti-digoxigenin 1/200 -Sigma- and anti-NCL 1/1000 -Abcam-) diluted in PBS 1X, 0.3% BSA, 0.1% saponine. The PLA was carried out using the Duolink PLA *in situ* kit, PLA probe anti-rabbit plus, the Duolink *in situ* PLA probe anti-mouse MINUS and the *in situ* detection reagent FarRed (all from Sigma) following the manufacturer’s protocol. PLA results were visualized using a Zeiss LSM780 confocal microscope. All the PLA experiments were performed at least three times independently and, each time, PLA dots were counted in 50–100 cells. For each PLA experiment, the following controls were used: w/o mRNA probe, w/o antibodies and with the control sense probe.

### ^35^S methionine pulse-labeling

8 × 10^5^ cells were transiently transfected with 4 μg of 235GAr-OVA or OVA vectors and, 8 h later, NCL silencing was performed using 40 nM of NCL siRNA or control siRNA (as previously described). Forty hours after the transfection, cells were incubated 30 min in a methionine-free medium. After incubation, 25 μM of MG132 proteasome inhibitor was added to the medium and cells were incubated for 45 min. Cells were then cultured in a medium containing 0.15 mCi ml^–1 35^S-methionine (Perkin Elmer, Boston, USA) for 1 h and collected. Cell pellets were suspended in 20 mM Tris-HCl, pH 7.5, 150 mM NaCl, 1% Igepal and treated as described above. Lysates were precleared with 1 μg normal rabbit serum (Dako) bound to protein G-Sepharose magnetic beads (GE Healthcare) for 30 min at 4 °C and further immunoprecipitated with 1 μg of anti-OVA polyclonal antibody (Sigma) or IgG-rabbit (Dako), prebound to protein G-Sepharose magnetic beads overnight at 4 °C. Beads were then washed and proteins eluted using 2X SDS loading buffer. Immunoprecipitates were analysed by SDS-PAGE using 10% precast NUPAGE gels (invitrogen). The amount of radiolabelled proteins was visualized using a Storm Phosphorimager (GE Healthcare).

### T-cell proliferation assay

Naive OVA_257–264_ specific CD8+ T cells were isolated by negative selection from peripheral and mesenteric lymph-nodes of 12-week-old female OT1 mice using the CD8^+^ T-cell isolation kit (Miltenyi Biotec, Germany). Afterwards, CD8^+^ T cells were stained with CellTraceTM Violet (Thermo Fisher Scientific, USA) according to the manufacturer’s protocol and mixed with H1299 cells cotransfected with mouse k^b^ expression vector and OVA or GAr-OVA constructs. For all the assays, 10^5^ H1299 cells were collected 48 h after transfection and co-incubated with 4 × 10^5^ stained T cells at 37 °C in humidified air/CO_2_ atmosphere in 1 ml of RPMI medium containing 10% FBS, 4 mM L-glutamine, 100 U ml^–1^ penicillin, 100 μg ml^–1^ streptomycin, 5 mM HEPES and 0.05 mM 2-mercaptoethanol (Sigma-Aldrich). After 3 days, cells were collected, stained with hamster anti-mouse CD3-APC (Miltenyi Biotec) and fixable viability dye eFluor 780 (eBioscience, USA) and analysed by FACS on a CANTO II flow cytometer (BD Biosciences, USA). Cells were gated for live CD3^+^ cells (10,000 events collected) and data were analysed using BD FACSDiva software version 8.0.1. The percentage of proliferating T cells was considered for statistical analysis.

### MTT assay

A total of 30,000 Mutu-1 cells were plated in 0.1 ml in 96-well flat bottom plates and exposed to PhenDC3 at the indicated concentrations or DMSO (vehicle). After 24 h, 10 μl of 5 mg ml^–1^ MTT solution (CT01-5, Merck Millipore) in PBS pH 7.4 was added to each well and incubated for 4 h. A volume of 0.1 ml of isopropanol-HCl 0.1 N-Triton X-100 10% was added to each well to dissolve the formazan crystals. The absorbance was then measured at 540 nm.

### Statistical analyses

Data shown are mean±s.d. of minimum three independent experiments. Two-tailed unpaired Student’s *t*-test was performed by comparing data to the corresponding reference point or as indicated and *P* values are shown. *P<0.05; **P<0.01; ***P<0.001; NS: not significant.

### Fluorescence displacement assay for G4 ligands

G4-fluorescence displacement (FID) assay is performed in a 96-well non-binding surface black with black bottom polystyrene microplates (Corning). Every ligand is tested on a line of the microplate, in duplicate (in other plate). The microplate is filled with (a) K^+^100 solution (qs for 200 μL) (b) 10 μl of a solution of prefolded oligonucleotides (5 μM) and TO (10 μM—2 molar equiv.) and (c) an extemporaneously prepared 5 μM ligand solution in K^+^100 buffer (0–100 μl along the line of the microplate, that is, from column A to column H: 0, 0.125, 0.25, 0.375, 0.5, 0.625, 0.75, 1.0, 1.25, 1.5, 2.0 and 2.5 μM). After 5 min of orbital shaking at 500 r.p.m., fluorescence is measured using the following experimental parameters; positioning delay: 0.5 s, 20 flashes per well, emission/excitation filters for TO: 485/520, gain adjusted at 80% of the fluorescence from the most fluorescent well (that is, a well from column A). The percentage of TO displacement is calculated from the fluorescence intensity (*F*), using: % TO displacement=1–*F*/*F*_0_ (*F*_0_ being the fluorescence from the fluorescent probe bound to DNA without added ligand. The percentage of displacement is then plotted as a function of the concentration of added ligand. The DNA affinity is evaluated by the concentration of ligand required to decrease the fluorescence of the probe by 50%, noted DC_50_, and determined after nonlinear fitting of the displacement curve.

### Data availability

The authors declare that data supporting the findings of this study are available within the article and the Supplementary Information, or available from the corresponding author upon reasonable request.

## Additional information

**How to cite this article:** Lista, M. J. *et al*. Nucleolin directly mediates Epstein-Barr virus immune evasion through binding to G-quadruplexes of EBNA1 mRNA. *Nat. Commun.* **8,** 16043 doi: 10.1038/ncomms16043 (2017).

**Publisher’s note:** Springer Nature remains neutral with regard to jurisdictional claims in published maps and institutional affiliations.

## Supplementary Material

Supplementary Information
