# Supplementary material for: Nucleolin directly mediates Epstein-Barr virus immune evasion through binding to G-quadruplexes of EBNA1 mRNA
Source: Nat Commun. 2017 Jul 7;8:16043. doi: 10.1038/ncomms16043 (PMC5504353; doi:10.1038/ncomms16043)
Supplement: Supplementary Information [file ncomms16043-s1.pdf]

Supplementary figure 1

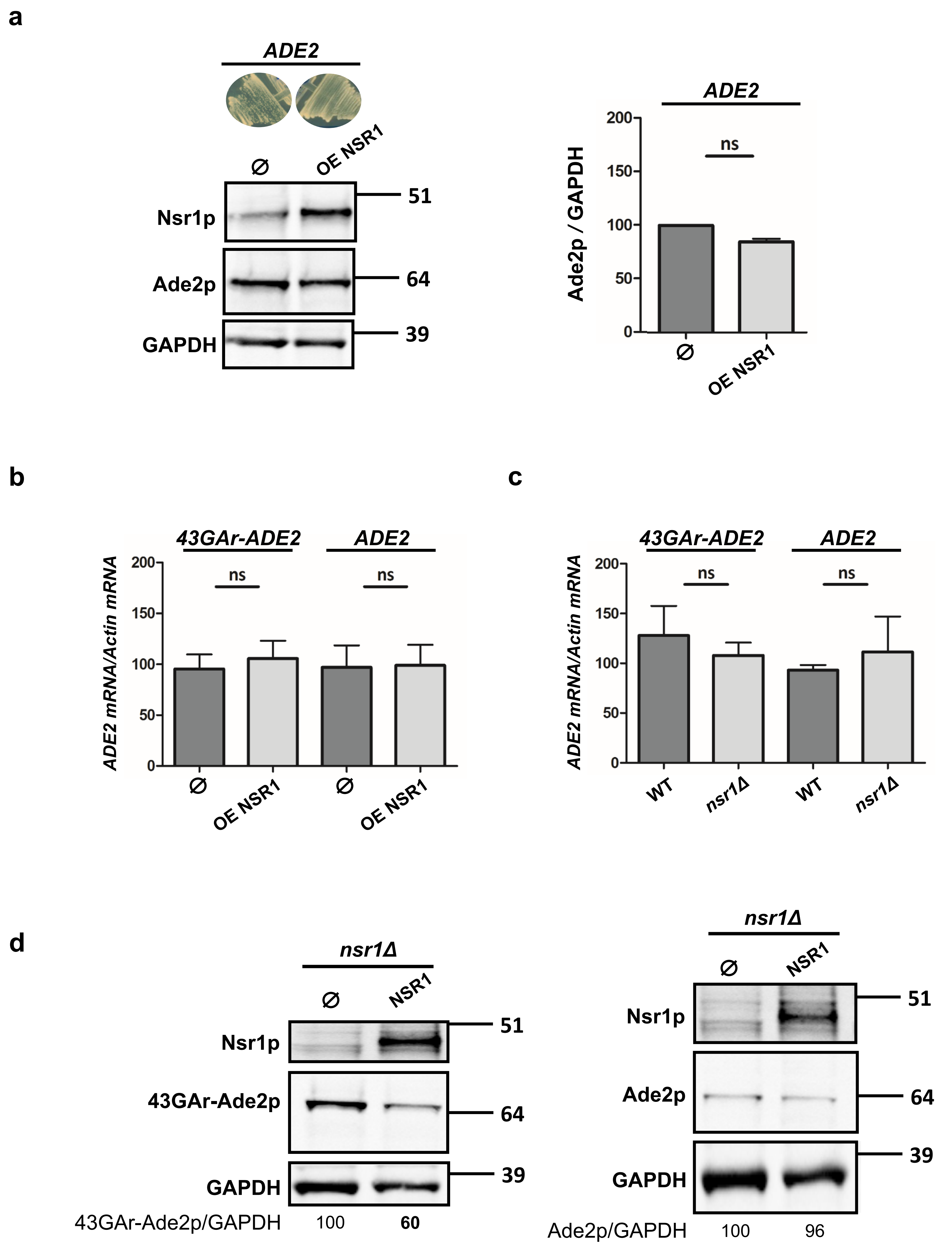

Supplementary Figure 1: Identification and confirmation of the critical role of nucleolin in GAr-based translation inhibition in yeast.

(a) Effect of *NSR1* overexpression on Ade2p level. The overexpression of *NSR1* gene, which encodes the yeast nucleolin, has no effect on the white color of *ADE2* expressing yeast cells and on the Ade2p protein level as evidenced by SDS-PAGE and western blot analysis (left panel). GAPDH was used as a loading control. The mean Ade2p/GAPDH ratios from 3 independent experiments are shown in the right panel and the results compared using the Student's *t*-test (ns: not significant).

(b) Overexpression of *NSR1* has no effect on *43GAr-ADE2* and *ADE2* mRNA level in yeast. Relative levels of *43GAr-ADE2* or *ADE2* mRNA as compared to actin mRNA in *NSR1*-overexpressing cells were determined by quantitative RT-PCR. The results were compared using the Student's *t*-test (ns: not significant).

(c) Deletion of *NSR1* has no effect on *43GAr-ADE2* and *ADE2* mRNA level in yeast. Relative levels of *43GAr-ADE2* or *ADE2* mRNA as compared to actin mRNA in *WT* or *nsr1Δ* cells were determined by quantitative RT-PCR. The results were compared using the Student's *t*-test (ns: not significant).

(d) Complementation of *NSR1* deletion by the *NSR1* gene expressed from a plasmid. SDS-PAGE and western blot analysis of the yeast *nsr1Δ* strain expressing 43GAr-Ade2p (left) or Ade2p (right) and expressing (right lanes), or not (left lanes), yeast *NSR1*. GAPDH was used as a loading control. The 43GAr-Ade2p/GAPDH or Ade2p/GAPDH ratios are indicated below the gels.

Supplementary figure 2

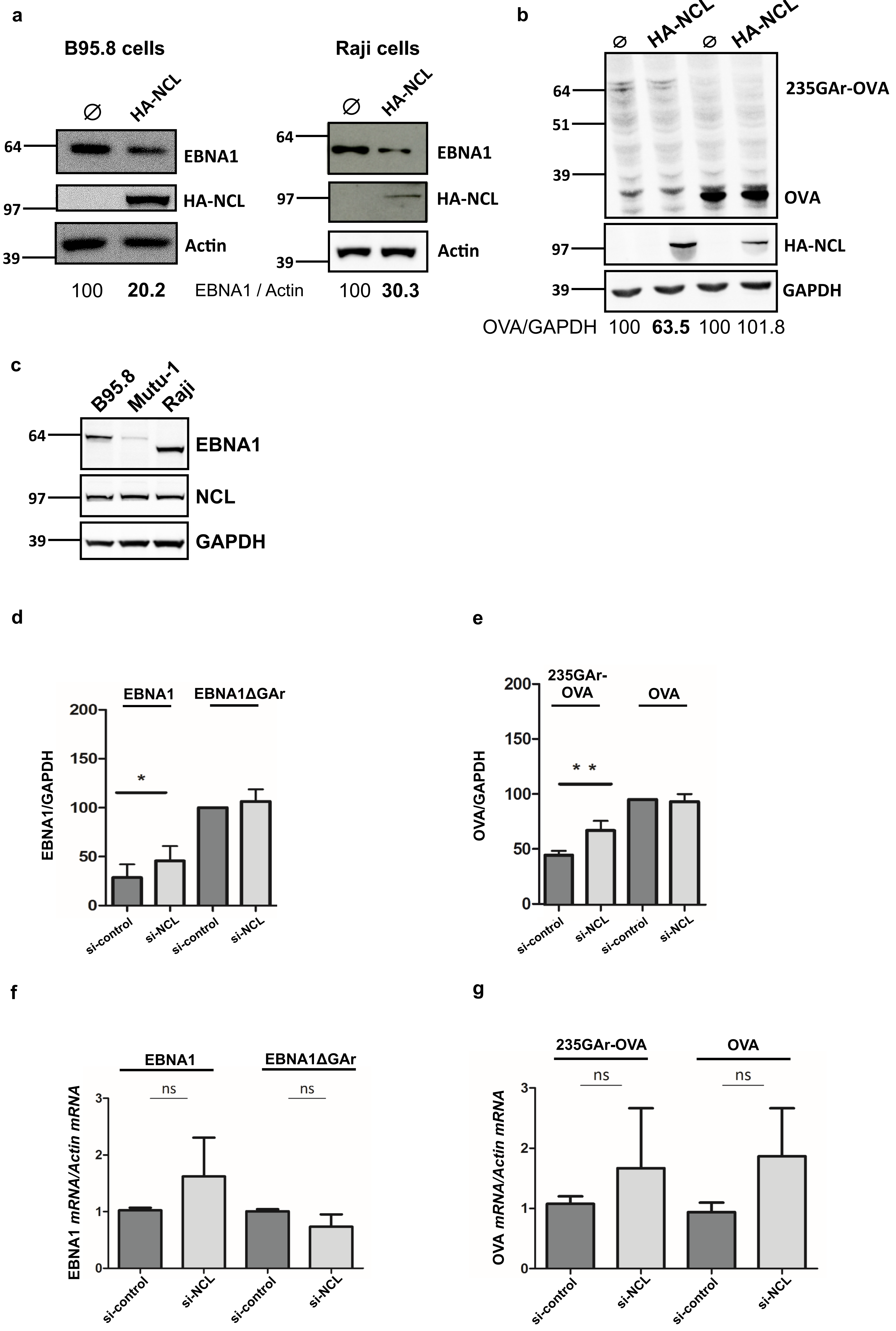

Supplementary Figure 2: Overexpression of NCL exacerbates GAr effect on protein expression whereas its downregulation suppresses its inhibitory effect on translation.

(a) SDS-PAGE and western blot analysis of the level of endogenous EBNA1 in two EBV-infected B cell lines (B95.8 and Raji as indicated) overexpressing (right lanes), or not (left lanes), HA-tagged nucleolin (HA-NCL). Actin was used as a loading control. EBNA1/actin ratios are indicated below the gels. Blots represent n≥3.

(b) SDS-PAGE and western blot analysis of the level of 235GAr-OVA or OVA in response to the overexpression of NCL. HCT116 cells were transfected with 235GAr-OVA or OVA and with NCL plasmids, as indicated. GAPDH was used as a loading control. The OVA/GAPDH or 235GAr-OVA/GAPDH protein level ratios are indicated below the gels. Blot represents n≥3.

(c) SDS-PAGE and western blot analysis of the level of endogenous EBNA1 and NCL in the three EBV-infected B cell lines used in this study. GAPDH was used as a loading control.

(d) Quantification of the experiments shown in **Figure 2c.** The mean EBNA1/GAPDH and EBNA1ΔGAr/GAPDH protein level ratios from 3 independent experiments are shown. The results were compared using the Student's *t*-test (**p*<0.05).

(e) Quantification of the experiments shown in **Figure 2d.** The mean 235GAr-OVA /GAPDH and OVA/GAPDH protein level ratios from 3 independent experiments are shown. The results were compared using the Student's *t*-test (***p*<0.01).

(f) siRNA-mediated NCL knockdown has no effect on EBNA1 and EBNA1ΔGAr mRNA level in H1299 cells. H1299 cells were transfected with EBNA1 or EBNA1ΔGAr and with control siRNA or siRNA against NCL, as indicated. Relative levels of EBNA1 or EBNA1ΔGAr mRNA as compared to actin mRNA in cells treated with siRNA targeting NCL or control siRNA as indicated were determined by quantitative RT-PCR. The results were compared using the Student's *t*-test (ns: not significant).

(g) Same experiment as in **(f)** except that H1299 cells were transfected with chicken ovalbumin (OVA) or 235GAr-OVA whose relative mRNA levels compared to actin mRNA were assessed

by quantitative RT-PCR. The results were compared using the Student's *t*-test (ns: not significant).

Supplementary figure 3

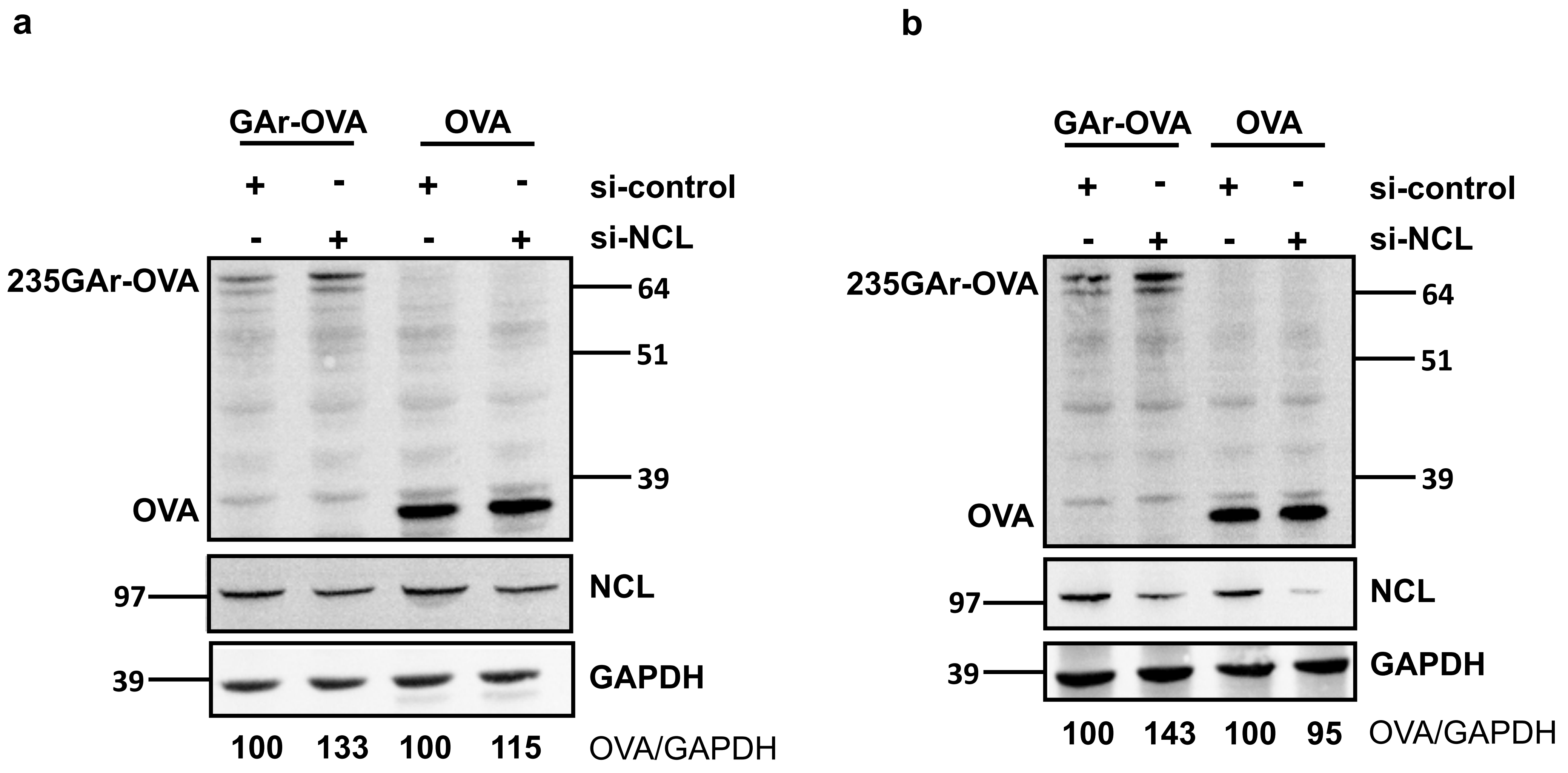

Supplementary Figure 3: NCL downregulation activates antigen presentation and recognition by T lymphocytes.

(a) SDS-PAGE and western blot analysis of the H1299 cells used in **Figure 3a** & **b**.

(b) SDS-PAGE and western blot analysis of the H1299 cells used in **Figure 3c** & **d**.

Supplementary figure 4

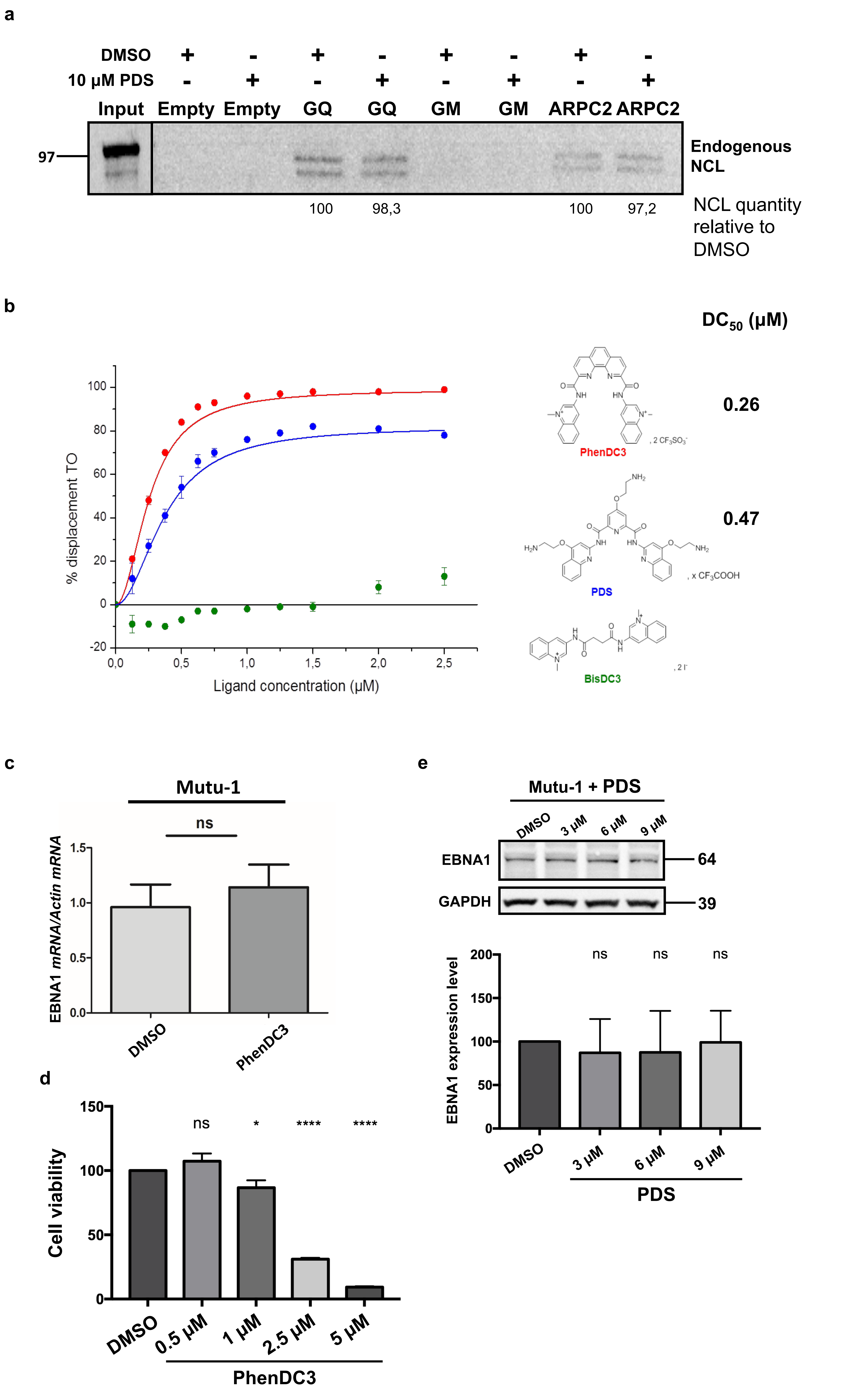

Supplementary Figure 4: PDS does not prevent NCL binding to both GAr's and *ARPC2* G4s and has a lower affinity than PhenDC3 for GAr's G4.

(a) Same experiment than in **Figure 4b** except that DMSO (vehicle) or PDS were added as indicated.

(b) HT-G4-FID Plots for PhenDC3 and PDS (see **Methods** section for description of the assay). The ability of both compounds to displace thiazole orange (TO) was assessed. Bis-DC3 was used as a negative control. The DC_{50} calculated from the obtained curves are indicated on the right.

(c) Treatment with 1 µM PhenDC3 has no effect on EBNA1 mRNA level in Mutu-1 cells. Relative levels of EBNA1 mRNA as compared to actin mRNA in PhenDC3- or DMSO-treated cells were determined by quantitative RT-PCR. The results were compared using the Student's *t*-test (ns: not significant).

(d) Toxicity of various concentration of PhenDC3 on Mutu-1 cells was assessed using MTT assay. The results obtained with the various concentrations of PhenDC3 were compared to the result obtained with cells treated by DMSO (compound vehicle) using the Student's *t*-test (*p<0.05; ***p<0.001; ns: not significant).

(e) PDS has no effect on endogenous EBNA1 level in Mutu-1 cells. Same experiment than in **Fig. 6e**, except that Mutu-1 cells were treated with 3, 6 or 9 µM PDS. The results were compared using the Student's *t*-test (ns: not significant).
